# Can You Hear What’s Coming? Failure to Replicate ERP Evidence for Phonological Prediction

**DOI:** 10.1162/nol_a_00078

**Published:** 2022-09-22

**Authors:** Victoria R. Poulton, Mante S. Nieuwland

**Affiliations:** Max Planck Institute for Psycholinguistics, Nijmegen, The Netherlands; MRC Cognition and Brain Sciences Unit, University of Cambridge, Cambridge, UK; Donders Institute for Brain, Cognition and Behaviour, Nijmegen, Radboud University, The Netherlands

**Keywords:** phonological mismatch negativity, phonological mapping negativity, N200, N400, spoken language comprehension, word form prediction, spoken word recognition

## Abstract

Prediction-based theories of language comprehension assume that listeners predict both the meaning and phonological form of likely upcoming words. In alleged event-related potential (ERP) demonstrations of phonological prediction, prediction-mismatching words elicit a phonological mismatch negativity (PMN), a frontocentral negativity that precedes the centroparietal N400 component. However, classification and replicability of the PMN has proven controversial, with ongoing debate on whether the PMN is a distinct component or merely an early part of the N400. In this electroencephalography (EEG) study, we therefore attempted to replicate the PMN effect and its separability from the N400, using a participant sample size (*N* = 48) that was more than double that of previous studies. Participants listened to sentences containing either a predictable word or an unpredictable word with/without phonological overlap with the predictable word. Preregistered analyses revealed a widely distributed negative-going ERP in response to unpredictable words in both the early (150–250 ms) and the N400 (300–500 ms) time windows. Bayes factor analysis yielded moderate evidence against a different scalp distribution of the effects in the two time windows. Although our findings do not speak against phonological prediction during sentence comprehension, they do speak against the PMN effect specifically as a marker of phonological prediction mismatch. Instead of an PMN effect, our results demonstrate the early onset of the auditory N400 effect associated with unpredictable words. Our failure to replicate further highlights the risk associated with commonly employed data-contingent analyses (e.g., analyses involving time windows or electrodes that were selected based on visual inspection) and small sample sizes in the cognitive neuroscience of language.

## INTRODUCTION

Many researchers in the field of cognitive neuroscience have endeavoured to identify the time course and neural signatures of linguistic prediction and comprehension. One approach involves studying components of event-related potentials (ERPs), as the timing and amplitude of these effects have historically given important insights into specific cognitive mechanisms and processes. However, the nature of certain prediction-related effects remains controversial. In the present study, we focused specifically on the phonological mismatch negativity (PMN; Connolly & Phillips, 1994; D’Arcy et al., 2004; Desroches et al., 2009; Newman et al., 2003) and the related N200 (Van den Brink et al., 2001; Van den Brink & Hagoort, 2004), which have been associated with predictions of spoken word form (phonological predictions). Several studies report prediction-related PMN effects, but their characterization and differentiation from the N400 have been inconsistent (for a review, see Nieuwland, 2019). This inconsistency undermines the pre-N400 negative component as a marker of phonological prediction—distinct from the N400 marker of semantic prediction—during sentence comprehension. The present study therefore addresses the following question: Is there a distinct neural effect that is elicited in response to violations of form predictions?

### The PMN and Phonological Prediction

The strongest stances on linguistic prediction propose that predictions occur at all levels of representation (Altmann & Mirković, 2009; Dell & Chang, 2014; Pickering & Gambi, 2018). While evidence for semantic prediction is well-established, the evidence for form (phonological/orthographic) prediction is less consistent. Studies of phonological prediction vary greatly with respect to their paradigms, the naturalness of the stimuli, and the behavioral and neural effects they have investigated. For example, the simplest paradigms for studying phonological prediction are those investigating the mismatch negativity (MMN) in oddball paradigms, where a deviant phoneme or speech segment shows the MMN effect (e.g., Näätänen et al., 1997). The magnitude of the MMN has been interpreted as an index of the magnitude of prediction error, the difference between the prediction and received input (Garrido et al., 2009).

However, paradigms that involve predictions between linguistic units at the same level, such as syllable- or word-priming experiments (within-level predictions), are not reflective of more naturalistic speech listening conditions. In contrast, during spoken sentence comprehension, phonological predictions about specific words would have to emerge from more general predictions about upcoming meaning, based on preceding semantic context. So, while phonological predictions are most often reported by studies that test within-level predictions, is phonological prediction a consistent phenomenon during sentence processing?

The answer to this question is not particularly clear. Studies that have attempted to disentangle effects of form and semantic prediction show inconsistent results. For example, DeLong et al. (2005) report an increased amplitude at the N400 for English articles (*a*/*an*) that are unpredictable based on expectations about whether the upcoming word should begin with a vowel or consonant. Although DeLong et al. interpreted these results as evidence for phonological form prediction, other studies, including a large-scale replication study, suggest that such N400 effects are not very reliable and are hard to detect (Ito et al., 2017; Nieuwland et al., 2018; but see also Ito et al., 2020, for a related manipulation in Italian).

Here, we turn to the phonological mismatch/mapping negativity (PMN; Connolly & Phillips, 1994) as a possible index of phonological prediction during spoken sentence processing. The PMN is sometimes referred to as the phonological mapping negativity in order to distinguish it from the mismatch negativity (MMN). However, there is also considerable debate over whether the PMN is distinguishable from the MMN, which has similar properties such as a frontocentral distribution and a sensitivity to phonological and acoustic manipulations (for a review, see Näätänen et al., 2007). Some authors state that the PMN and MMN should not be conflated (e.g., Archibald & Joanisse, 2011; Connolly & Phillips, 1994) while others provide evidence for the MMN within sentence processing paradigms (e.g., Boulenger et al., 2011; Signoret et al., 2020).

The PMN is characterized separately from the N400, which is often interpreted as an index of semantic processing (Kutas & Federmeier, 2011). The PMN effect was first reported by Connolly and Phillips (1994) as a negative voltage deflection with a fronto-central distribution that peaks in the 150–350 ms time window after word onset, in contrast to effects in the time window typically associated with the N400 (i.e., 300–500 ms after word onset). Connolly and Phillips reported that in sentences such as “Don caught the ball with his *hand*/*glove*,” the word *glove* elicits a fronto-centrally distributed PMN but not an N400 effect. Their explanation for this pattern was that *glove* was a semantic match (i.e., similar in meaning to) but a phonological mismatch with the predictable word *hand*. Conversely, only an N400 effect was elicited by words like *luggage* in sentences such as “The gambler had a streak of bad *luck*/*luggage*,” which is semantically unacceptable but whose initial sounds match that of the expected word *luck*. From these patterns, they concluded that the PMN indexes detection of a mismatch between an incoming sound and a phonological representation of a highly predictable word. Moreover, they concluded that phonological mismatch detection is independent of and fully precedes the semantic processes indexed by the N400.

Further support for the PMN comes from paradigms that do not involve sentences and instead use isolated words or sounds. One example is the phoneme deletion task, where participants are asked to predict what an input word would sound like after removing the word-initial sound(s) (e.g., given the word *clap*, participants were asked to remove the first sound, predicting *lap*). In this task, prediction-mismatching sounds elicit a PMN effect (Newman et al., 2003). This PMN effect was observed regardless of their phonological overlap with the expected stimulus, consistent with an “all-or-none” response. The PMN has also been elicited by mismatch between a spoken word and a visual object, for example when participants heard *bone* but had viewed an image of a “cone” (Desroches et al., 2009). In a similar task, an increased PMN is reported for words that contain coarticulatory (i.e., subphonemic) cues that mismatch with the word associated with the visual image. For example, following the presentation of an image of a “hat,” an increased PMN is reported for words beginning with an acoustically manipulated /h/ containing coarticulatory cues for a different vowel (Archibald & Joanisse, 2011).

### Is the PMN Distinct from the N400?

Despite previous support for the PMN as an ERP index of phonological prediction during sentence comprehension, we remain skeptical of this interpretation of the PMN (for a detailed review, see Nieuwland, 2019; related arguments were raised by Diaz & Swaab, 2007; Groppe et al., 2010; Van Petten et al., 1999). One important reason for this skepticism is that the definition and, therefore, the manifestation of the PMN effect has been very inconsistent throughout the literature. For example, the claimed PMN effect of Connolly and Phillips (1994) was obtained by selecting the most negative peak within the 150–350 ms time window from the ERPs in each condition. The problem with this effect definition is its sensitivity to random fluctuations (noise) in the ERP signal and that it will obtain a PMN effect even in absence of any evident component peaks. Furthermore, any voltage difference in this time window is taken to demonstrate the PMN effect. This is problematic in light of evidence for modality differences in the timing and duration of the N400, with the auditory N400 showing an earlier onset and a sustained duration compared to the visual N400 in a semantic priming task (Holcomb & Neville, 1990). Therefore, this method ignores the possibility that the voltage difference comes from the rising trajectory of the onset of the N400 activity. This serves as a reminder that time windows alone are not diagnostic of distinct ERP components.

Several subsequent studies also relied on visual inspection but concluded that not only mismatching conditions but also the matching condition elicits a negative deflection, and called this deflection the N200. For example, Van den Brink and colleagues (2001) presented participants Dutch sentences such as *De schilder kleurde de details in met een klein penseel* / *pensioen* / *doolhof* (The painter coloured the details with a small paint brush / pension / labyrinth), where the final word was either congruent (*penseel*), initially congruent (*pensioen*, overlapping in word onset with the congruent completion), or fully incongruent (*doolhof*). They observed an N200 in all three conditions that differed in amplitude. They concluded that the N200 reflects a more general process, namely one at the interface of activated lexical forms and contextual meaning (i.e., when the context begins to play a role in lexical selection). According to this interpretation, the processes implicated in the N200 are at a later stage of lexical selection following phonological analysis, while the processes implicated in the PMN interpretation reflect the early stages of lexical access, specifically the comparison of phonological expectations and input.

Van den Brink et al. (2001) distinguished the N200 from the N400 on visual inspection based on temporal characteristics and also using scalp topographies. This was important because like the PMN, the N200 reportedly has a more frontal or more uniform distribution (i.e., equally large at anterior and posterior channels) than the more posterior N400 (e.g., Connolly & Phillips, 1994; Van den Brink et al., 2001). However, a subsequent follow-up study by Van den Brink and Hagoort (2004) did not find a different scalp distribution for the N200 and N400. Like with the PMN, it is therefore not clear whether the N200 is genuinely distinct from the N400, in timing or topography.

Yet other studies have not observed an early negative deflection for mismatching words at all, raising further questions about the reliability of the PMN and N200 effects. For example, Van Petten et al. (1999) presented participants with spoken sentences such as *It was a pleasant surprise to find that the car repair bill was only seventeen*. … The sentences could end with a word in one of four possible conditions: congruent (*dollars*), which was semantically predictable; rhyme-overlapping (*scholars*); onset-overlapping (*dolphins*); or completely anomalous (*bureaus*). For rhyme-overlapping and anomalous words, negative-going waveforms diverged earlier than those time-locked to the onset-overlapping word. This is similar to what is reported in studies of the N200/PMN. However, this early difference between the conditions did not lead to a change in the peak latency of the N400. Furthermore, no N400 difference was observed between the rhyme-overlapping and the completely anomalous conditions, which is inconsistent with the hypothesis that participants generated a phonological template to match against the input. Van Petten et al. (1999) concluded that the early negativity is an early onset to the N400, reflecting the detection of a mismatch between the meaning of the auditory input and the semantic expectation, a process that is facilitated by a rapid mapping of input to semantics.

A related study investigated the ERPs associated with violations of expectations in both spoken sentences and alliterative word lists (Diaz & Swaab, 2007). While they observed an early negativity for the mismatching words in the alliterative lists, ERPs in the sentence processing experiment were more typical of the N400, without a distinct effect resembling the previously reported N200/PMN. The authors argue that this may indicate that phonological and semantic processing are differentially engaged between the two tasks, resulting in different effects in the ERPs (Diaz & Swaab, 2007). Whether it is the case that the early negativities reflect an early onset to the N400, or that multiple processes underlie the N400 and are differentially engaged in different tasks, the lack of evidence for a distinct N200/PMN in these studies is reason to call into question the reliability of these early components.

Finally, as argued by Nieuwland (2019), what appears to be peak in the ERP may actually be residual alpha activity (i.e., alpha activity that is not related to the manipulation of interest). Alpha waves are sometimes evident in the raw electroencephalography (EEG) data, in particular when participants close their eyes, when there are no or few visual stimuli, or when the participant is less alert. As alpha oscillations correspond to frequencies in the range around 10 Hz, residual alpha activity in the average ERP could show up as a peak roughly every 100 ms. Such alpha activity is clearly visible in the different ERP studies to date. Thus, it is possible that the peak preceding the N400 is the result of residual alpha activity and has been mislabeled as a distinct component. We also suspect that residual alpha may emerge in the auditory comprehension experiments as opposed to visual comprehension experiments because the participants are less visually engaged in the absence of a serial presentation of written words. Residual alpha should disappear with sufficient data (i.e., if residual alpha is indeed noise and entirely unrelated to the manipulation of interest, averaging over a large enough number of trials would cancel out such residual alpha activity). However, many of these studies have relatively small sample sizes (*N* < 30). Therefore, if an analysis approach only concerns the most negative point in the waveform in a specific time window, researchers could be mistakenly identifying a peak due to alpha as a component peak.

In sum, we have highlighted the methodological issues with the PMN and N200, pointing at inconsistencies in how these early components are defined and whether they are even observed consistently across studies. Taken together, evidence for early components that are distinct from the N400 is questionable.

### The Present Study

To address these issues, the present study investigated whether unpredictable words elicit a distinct PMN or N200 effect compared to predictable words, or whether they merely elicit an auditory N400 effect. For simplicity, we refer to this early component as the N200/PMN throughout this article, since the corresponding time windows and topographic descriptions are very similar. However, we are not conflating the theoretical interpretations of the PMN versus the N200.

Participants were presented with spoken sentences ending in either predictable or unpredictable final words while we recorded continuous EEG activity. Two observations are critical to support a distinct early negative ERP component: First, responses to unpredictable words must differ from predictable words in the early time window; and second, the scalp distribution of this early effect must differ from the scalp distribution of the effect observed in the typical N400 time window. If there is a distinct early negativity that is sensitive to violations of phonological expectations, it is expected that this will have a more frontal and widely distributed scalp topography when compared to the N400.

We were also interested in the proposed all-or-none response of the N200/PMN to phonological mismatch (e.g., Newman et al., 2003). To investigate this, we manipulated the amount of phonological mismatch in the first syllable of the unpredictable conditions (see Table 1). Two unpredictable words were selected corresponding to the two unpredictable conditions: *partial overlap*, in which the first consonant of the unpredictable word conflicts with the first consonant of the predictable word, while the following phonemes in the first syllable overlap; and *no overlap*, in which unpredictable and predictable words do not have phonological overlap in the first syllable. An observed difference in ERP amplitude between the different unpredictable conditions would provide evidence against this all-or-none account, indicating that listeners engage in a phonological matching process. We note here that our paradigm is most similar to that of Van den Brink et al. (2001), who used three different conditions (fully congruent, initially congruent, and fully incongruent). Our paradigm differs from Van den Brink et al. (2001) in the location of any phonological overlap: Our unpredictable conditions (partial overlap and no overlap) are unpredictable in their first phoneme and overall semantics compared to our predictable condition, whereas their initially congruent condition overlaps in first phoneme and differs in overall semantics from their fully congruent condition. For example, our predictable *boeket* (‘bouquet’) is replaced with *toetertje* (‘horn’), while their fully congruent *penseel* (‘paint brush’) is replaced with initially congruent *pensioen* (‘pension’). Therefore, our paradigm still maintains conditions with some phonological overlap between predictable and unpredictable words, as does theirs.

**Table 1.** Example experimental items with English translations.

**Sentence frame**	**Critical words**			**Target consonants**	
	**Predictable**	**Partial overlap**	**No overlap**	**Predictable**	**Unpredictable**
De bloemist bezorgde bij Lisa een mooi	boeket	toetertje	tonnetje	/b/	/t/
The florist delivered to Lisa a beautiful	bouquet	horn	barrel		
Het nachtdier kon erg goed zien in het	donker	tonnetje	toetertje	/d/	/t/
The nocturnal animal could see very well in the	dark	barrel	horn		

## MATERIALS AND METHODS

### Preregistration

We preregistered the design, EEG recording, preprocessing, and analysis procedures on the Open Science Framework (OSF; project name “Phonological processing during sentence comprehension”; https://osf.io/a4bpw/). Not preregistered analyses will be referred to as exploratory.

In accordance with the Peer Reviewers’ Openness Initiative (https://opennessinitiative.org, Morey et al., 2016), all materials (stimuli, data, scripts, and figures) associated with this manuscript were available during the review process and remain available on OSF project “Phonological processing during sentence comprehension” at https://osf.io/a4bpw/.

### Participants

In total, there were 73 participants in the experiment, all of whom were native speakers of Dutch. Our statistical analysis was based on a preregistered sample size of *N* = 48, which we reached after replacing data from 3 participants due to technical issues, and replacing the data from 22 participants because they did not meet our (fairly strict) exclusion criteria (see the section EEG recording and preprocessing). No participants were excluded on the basis of their performance on the comprehension questions as all achieved an accuracy greater than 80% (mean = 98%, *SD* = 2.2%). All participants were recruited from the subject pool at the Max Planck Institute for Psycholinguistics in Nijmegen, The Netherlands, and were paid for their time. We recruited participants aged 18–40 years who self-reported no history of hearing impairments or neurological impairments. Written consent was obtained in accordance with ethics approval by the Ethics Committee for Behavioral Research of the Social Sciences Faculty at Radboud University Nijmegen in compliance with the Declaration of Helsinki.

### Materials

#### Experimental items

The initial set of experimental items consisted of 240 Dutch sentence frames, the majority of which were taken from Van den Brink et al. (2001). Predictable words began with a single consonant or consonant cluster, but the first phoneme was always one of four target phonemes (/b/, /d/, /p/, or /t/). All target phonemes were plosives. The release burst of plosives provides a clear physical marker for time-locking the ERPs, which may benefit the detection of small, time-locked ERP effects.

All experimental sentences were submitted to a web-based sentence completion test or *cloze procedure* (Taylor, 1953). The test was completed by 22 participants, none of whom participated in the main EEG experiment. Participants were presented with the 240 sentence frames with the final word omitted. They were asked to read the frame and complete the sentence with the first word that came to mind.

We calculated cloze probability as the proportion of responses that matched our intended target. Sentences with a minimum cloze probability of 0.7 were selected from this cloze test for use in the EEG experiment. This cloze probability is relatively high compared to other studies, some of which had minimum cloze probability of 0.5 (e.g., Van den Brink et al., 2001). In the case of a 0.5 cloze probability, it is possible that another candidate is equally likely to be predicted as the presumed target. Thus, we chose the probability of 0.7 in order to limit the number of predictable candidates that differed from our target. In addition, we selected 20 sentences from a previous cloze procedure (Fleur et al., 2020). The final set of experimental items included 210 sentences with a mean cloze probability of 0.92.

We created semantically anomalous sentences by replacing the sentence-final, predictable word with an unpredictable word that also began with one of the four target plosives (/b/, /d/, /p/, or /t/). Unpredictable words were not used as predictable words in any of the sentences, but did occur in either of the unpredictable conditions—once as a partial overlap word and once as a no overlap word—in order to ensure that the average lexical frequency of the unpredictable words was the same for both conditions. A single participant would only see one version of the sentence frame, ending with a word from one of the three conditions, and never saw the same target word twice.

#### Filler sentences

We added 140 filler items that were not semantically anomalous. We did this to increase the number of semantically non-anomalous sentences in the experiment. The final set consisted of 60% non-anomalous and 40% anomalous sentences. These filler sentences were taken from sets of items that were previously normed with a cloze probability test (Fleur et al., 2020; Van den Brink et al., 2001) and were thus not included in the cloze test outlined above. The filler sentences varied in length in order to prevent the participants from becoming too familiar with the materials. The final word of each of the filler sentences began with a phoneme that was not one of the critical phonemes (i.e., not /b/, /d/, /p/, or /t/).

#### Stimulus recordings

The final item set included 630 sentences (210 × 3) and 140 fillers for a total of 770 recorded items. Recordings were made by a female native speaker of Dutch in a noise attenuated booth and took place over five approximately one-hour sessions. The speaker was instructed to produce the sentences at a normal but not fast pace, with normal intonation and prosody. Sentences were produced with the final word included. In previous studies, sentence frames and critical words were recorded separately and then spliced together with a delay between the end of the sentence and the critical word (e.g., Van Petten et al., 1999). This method can ensure similarity across items and can facilitate time-locking to word onset. However, important coarticulatory information can be lost and the introduction of an artificial delay before the critical word is not reflective of natural speech (Diaz & Swaab, 2007). Recording the entirety of the sentence, including the final critical word, preserves coarticulation and natural prosody during production. We then scaled the intensity of the sentences to an average intensity of 70 dB using the PRAAT software (Boersma & Weenink, 2019).

Based on a suggestion from a reviewer, we conducted an additional acoustic analysis of the stimuli to investigate systematic differences in the recordings across conditions, as acoustic differences may act as a cue to the listener about the predictability of the upcoming sentence-final word. To summarize, while we did find some differences in the pitch of the contexts and relative intensity of the sentence-final words, these differences are not sufficient to explain our ultimate ERP findings. A full supplemental report can be found on the OSF page (https://osf.io/a4bpw/).

**Figure 1.**
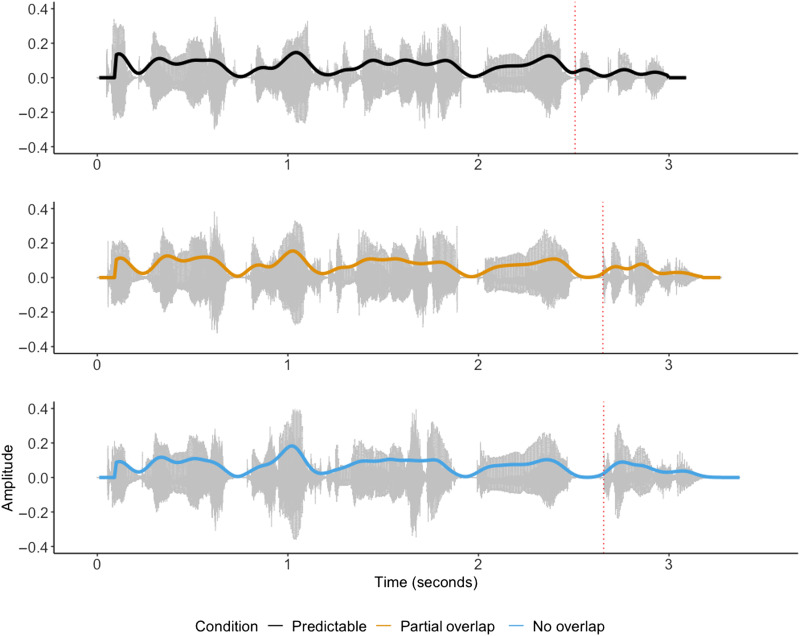
Acoustic waveforms and speech envelopes plotted for the example sentence *De bloemist bezorgde bij Lisa een mooi boeket* / *toetertje* / *tonnetje* (The florist delivered to Lisa a beautiful bouquet / horn / barrel). The panels correspond to the sentence in each condition, Predictable (black), Partial overlap (orange), and No overlap (blue), from top to bottom, respectively. Each sentence was recorded separately with the final word included in the initial recording. Vertical dotted lines indicate the timing of the critical words’ plosive burst used for event-related potential (ERP) time-locking.

Previous work on Dutch consonant perception suggests that the burst contains much of the information required for consonant identification (Smits et al., 2003; Warner et al., 2005). Indeed, the analysis by Van den Brink et al. (2001) used ERPs time-locked to the onset of the burst in the critical word. For the present study, we visually inspected the spectrograms of the recordings to identify the timing of the burst at the onset of each critical word, and we time-locked all reported ERPs to this word-onset point. Figure 1 provides a visualization of the acoustic waveforms and the time-locking points for the first set of example sentences in Table 1.

### EEG Experiment

#### Procedure

Participants were seated in front of two speakers and a monitor. They were instructed to listen to the sentences for comprehension and answer occasional comprehension questions. They were also instructed to fixate to a fixation point (the plus symbol “+”) in the center of the screen and to refrain from blinking and moving while the symbol was displayed.

We constructed three experimental lists with each containing 210 experimental sentences (70 for each condition) and arranged them such that no sentence frame occurred more than once. Each list also contained all 140 fillers for a total of 350 sentences per list. We divided the experiment into seven blocks of 50 sentences each. Each list began with the same five practice items, selected from the fillers. Comprehension questions followed approximately 25% of the sentences, with 88 yes/no questions distributed across the experimental blocks and 2 yes/no questions in the practice. Since the experiment was self-paced, participants could take breaks between sentences and longer breaks between blocks, resulting in EEG recording sessions that were 60–70 minutes in length.

#### EEG recording and preprocessing

We recorded EEG using BrainAmps amplifiers (Brain Products, Germany) at 1000 Hz and a standard 32-electrode actiCap montage (Brain Products, Germany) arranged according to the 10–20 system, with 27 out of the 32 electrodes mounted in the cap. From the remaining electrodes, we used two to measure vertical eye movements, two for horizontal eye movements, and one for the right mastoid. Additionally, we placed the reference electrode on the left mastoid and the ground electrode in the cap on the forehead.

We preprocessed data in BrainVision Analyzer (Version 2.1.2; https://www.brainproducts.com/). We first performed visual inspection to identify and interpolate “bad channels” (i.e., those with drift, spiking, or excess line noise), to a maximum of four channels. We then band-pass filtered the data between 0.1–30 Hz (24 dB/octave roll-off) and re-referenced offline to the mastoid-average. We then downsampled the data to 500 Hz and segmented the data into epochs from −500 ms before to +1250 ms after word onset.

We performed visual inspection to identify and reject “bad” trials (e.g., those with excess movement-related artifacts or muscle activity), and then corrected for blinks, eye movements, and steady muscle activity using independent component analysis. Subsequently, we performed baseline correction using a baseline window of −200 ms before the onset of the critical words, and applied automatic segment rejection to remove segments with voltages values exceeding a preregistered threshold of ±60 μV. We used this relatively strict threshold to minimize the effects of high amplitude alpha fluctuations on the results. Following our preregistration, we excluded participants retaining fewer than 55 trials on average per condition, and replaced those data by recruiting additional participants in order to meet our preregistered, final sample size of *N* = 48 for our statistical analysis.

### Analysis

We investigated the ERP amplitude in the two time windows of interest, namely an “early” N200/PMN time window (150–250 ms after word onset) and a typical N400 time window (300–500 ms). We analyzed the data in a predefined anterior (F3, F4, Fz, F7, F8, FCz, FC1, FC2, FC5, FC6) and a posterior (CP1, CP2, CP5, CP6, Pz, P3, P4, P7, P8) region of interest (ROI), averaging voltage across electrodes in each region. The time windows were identical to those used in Van den Brink et al. (2001) and Van den Brink and Hagoort (2004). We selected anterior and posterior electrode clusters that approximately matched those used by Van den Brink and colleagues.

We conducted a linear mixed-effects analysis using the lme4 package (Bates et al., 2015) and the lmertest package (Kuznetsova et al., 2017) in R (R Core Team, 2021) to investigate the average amplitude of the ERPs in the ROIs. The first model, Model 1 (Equation 1), included the factors of condition, ROI, and their interaction as fixed effects. Random slopes were fitted by-participant and by-item for the fixed effect of condition. Condition and ROI were deviation coded. As this model did not converge, the complexity was reduced to Model 2 (Equation 2), which included the same fixed effects as the first model, and random intercepts by-participant and by-item. This analysis was conducted in both the early N200/PMN time window and the N400 time window. Additional preregistered analyses included pairwise comparisons of the least-squares means across conditions in both the early and late time window using the emmeans() functions from the the emmeans package (Lenth, 2019).

Model 2 implausibly assumes that all subjects show the same effect of Condition and ROI (and likewise for items), and this type of model is associated with high type I error rates. To deal with this issue, we performed exploratory Bayesian mixed-effects model analyses with a fully maximal random effects structure (by-subject and by-item random intercepts and slopes for the main effect of Condition and ROI and their interaction). This analysis used the default, mildly informative priors from the brms package (Bürkner, 2017). We briefly compare the different analyses in the Results section and report the Bayesian analysis and full results in an Rmarkdown supplement on our OSF page (https://osf.io/a4bpw/).

To explore the distribution of the effects in the two time windows, we conducted model comparison between Model 2 (Equation 2) and Model 3 (Equation 3) below, which has the same random effect structure as Model 2 but does not include the Condition * ROI interaction. Model comparison allowed us to investigate whether the inclusion of the interaction provides a better fit of the data. Models were compared using the anova() function from the stats package (R Core Team, 2021), separately for both time windows. Based on the previous literature, we expected that there would be no interaction in the early N200/PMN time window, indicating a widely distributed effect. Furthermore, we expected an interaction in the late N400 time window, indicating a more posteriorly distributed effect.

$$\text{Model}\;1:\text{Voltage}∼\text{Condition}*\text{ROI}+\left(\text{Condition}|\text{Participant}\right)+\left(\text{Condition}|\text{Item}\right)$$ (1)

$$\text{Model}\;2:\text{Voltage}∼\text{Condition}*\text{ROI}+\left(1|\text{Participant}\right)+\left(1|\text{Item}\right)$$ (2)

$$\text{Model}\;3:\text{Voltage}∼\text{Condition}+\text{ROI}+\left(1|\text{Participant}\right)+\left(1|\text{Item}\right)$$ (3)

We also conducted a comparison of the scalp distributions in the two time windows following the procedure of Van den Brink et al. (2001). This involved *z*-transforming the difference scores between the no overlap and predictable conditions at each electrode per participant. These conditions were selected as they were analogous to the fully incongruent and congruent used by Van den Brink et al. (2001). Difference scores were calculated by subtracting the means of the conditions for each participant at each electrode and in each time window. The difference scores were then scaled separately for each time window in order to avoid a misinterpretation of amplitude difference between the two time windows as a distributional difference (McCarthy & Wood, 1985). To assess distributional differences, we then conducted an analysis of variance (ANOVA) with the fixed effects of Electrode and Time and their interaction, and an ANOVA with the fixed effects of ROI (using our predefined ROIs) and Time and their interaction, following the procedures of Van den Brink and colleagues.

In an exploratory analysis, we assessed the evidence in support of the ROI and Time Window interaction, with a Bayes factor analysis of the ROI * Time ANOVA using the BayesFactor package (Morey & Rouder, 2018).

## RESULTS

**Figure 2.**
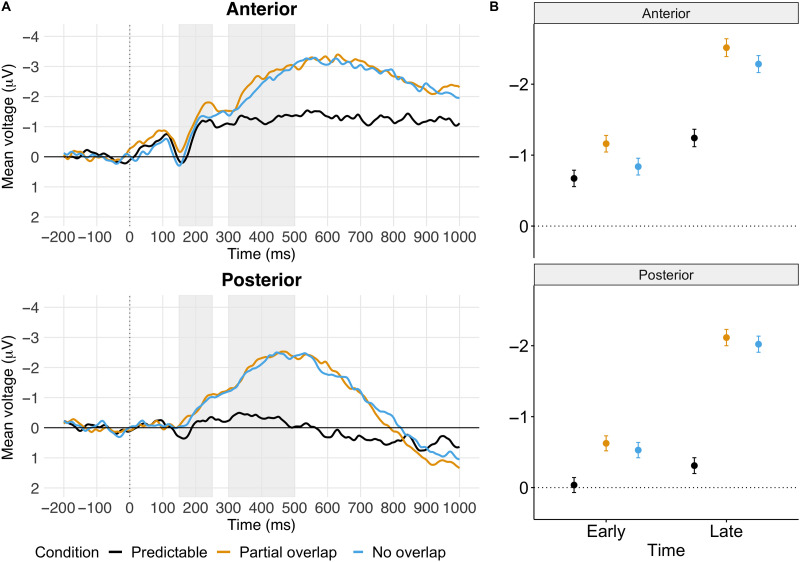
(A) Grand average ERPs for the anterior (upper left panel) and posterior (bottom left panel) regions of interest (ROIs). ERPs are time-locked to the burst of the word initial consonant. Grey vertical bars indicate the preregistered time windows of interest: the early (150–250 ms) time window where we tested for the N200/PMN, and the late (300–500 ms) time window where we tested for the N400. (B) Visualization of the mean voltage per condition in each ROI (Anterior vs. Posterior) and Time window (Early vs. Late) of interest.

**Figure 3.**
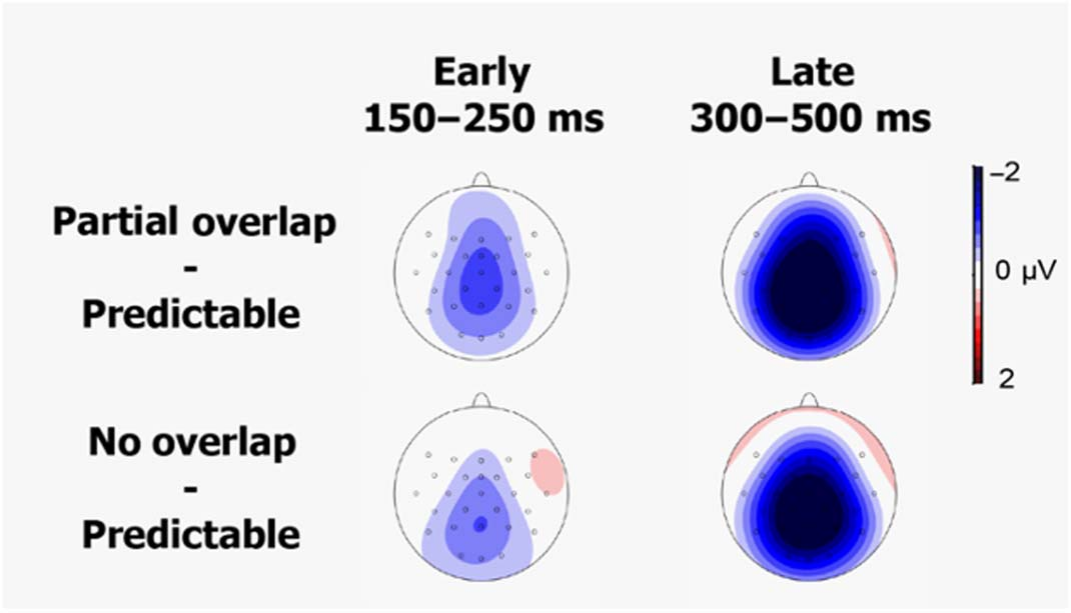
Scalp topographies of the difference waves between the partial overlap and the predictable condition, and the no overlap and the predictable condition, for both time windows of interest.

Grand average ERPs are displayed in Figure 2, and scalp topographies of difference waves are displayed in Figure 3. The waveforms of the unpredictable conditions diverge from the predictable condition at around 150 ms post stimulus. Both unpredictable conditions elicited an increased negative ERP amplitude (N400 effect).

### The Early Time Window (150–250 ms)

Voltage in the early time window did not yield a statistically significant interaction between condition and ROI (*χ* ^2^(2) = 2.29, *p* = 0.32; for details of the lmer results, see online Appendix B in the Supporting Information (located at https://doi.org/10.1162/nol_a_00078). Pairwise comparisons of the condition means are outlined in Table 2. In the Anterior ROI, partial overlap elicited more negative voltage compared to predictable words. In the Posterior ROI, no overlap and partial overlap elicited more negative voltage than predictable words. This pattern of pairwise comparisons was confirmed with an exploratory Bayesian analysis with a fully maximal random effect structure.

**Table 2.** Results of the pairwise comparisons of the least squares means in the early N200/PMN time window (based on Model 2).

**Contrast**	**ROI**	**Coefficient**	***SE***	**95% CI**	***t***	***p***
No overlap - Partial overlap	Anterior	0.32	0.16	(0.02, 0.63)	2.06	0.235
No overlap - Predictable	Anterior	−0.17	0.16	(−0.48, 0.14)	−1.08	>0.999
Partial overlap - Predictable	Anterior	−0.49	0.16	(−0.80, −0.19)	−3.14	**0.010**
No overlap - Partial overlap	Posterior	0.10	0.16	(−0.21, 0.40)	0.61	>0.999
No overlap - Predictable	Posterior	−0.50	0.16	(−0.80, −0.19)	−3.17	**0.009**
Partial overlap - Predictable	Posterior	−0.59	0.16	(−0.90, −0.28)	−3.77	**<0.001**

*Note*. Confidence level: 95%. Significant *p* values (<0.05) following Bonferroni correction for multiple comparisons are noted in **bold**.

### The Late Time Window (300–500 ms)

Voltage in the late time window yielded a significant interaction between condition and ROI (*χ* ^2^(2) = 9.19, *p* = 0.045; see online Appendix B for details). Pairwise comparisons of the condition means are outlined in Table 3. Voltage difference was most prominent at the posterior ROI, although in both ROIs voltage was more negative for no overlap and partial overlap compared to predictable words. There was no clear evidence for a difference between no overlap and partial overlap. Our exploratory Bayesian analysis confirmed the contrast estimates but yielded credible intervals that were somewhat wider than the 95% confidence intervals reported in Table 3. This Bayesian analysis did not support the condition by ROI interaction effect.

**Table 3.** Results of the pairwise comparisons of the least squares means in the late N400 time window (based on Model 2).

**Contrast**	**ROI**	**Coefficient**	***SE***	**95% CI**	***t***	***p***
No overlap - Partial overlap	Anterior	0.23	0.16	(−0.09, 0.55)	1.39	0.989
No overlap - Predictable	Anterior	−1.05	0.16	(−1.37, −0.73)	−6.37	**<0.001**
Partial overlap - Predictable	Anterior	−1.28	0.17	(−1.60, −0.95)	−7.74	**<0.001**
No overlap - Partial overlap	Posterior	0.09	0.16	(−0.23, 0.41)	0.55	>0.999
No overlap - Predictable	Posterior	−1.72	0.16	(−2.04, −1.40)	−10.43	**<0.001**
Partial overlap - Predictable	Posterior	−1.81	0.17	(−2.13, −1.49)	−10.96	**<0.001**

*Note*. Confidence level: 95%. Significant *p* values (<0.05) following Bonferroni correction for multiple comparisons are noted in **bold**.

### Scalp Distribution Analysis

We conducted a preregistered analysis of the spatial distribution of the ERP effects in the two ROIs and Time windows. Following Van den Brink et al. (2001), we *z*-transformed the difference scores between the no overlap and the predictable conditions, which were analogous to the fully incongruent and congruent conditions used by Van den Brink et al. We calculated the difference scores by subtracting the means of the conditions for each participant at all channels and in both time windows. We scaled the difference scores separately for each time window to ensure that differences in amplitude observed between the two time windows were not incorrectly interpreted as differences in distribution (McCarthy & Wood, 1985).

The first ANOVA included main effects of Channel (27-levels) and Time (2-levels, Early and Late). The results are outlined in Table 4. The effect of interest is the Channel * Time interaction, as this may indicate a difference in distribution between the two time windows. However, this interaction was not significant (*F*(26, 2538) = 0.998, *p* = 0.466).

**Table 4.** Results of the Channel(27) * Time(2) ANOVA on the *z*-transformed voltage difference between the no overlap condition and the predictable condition.

**Parameter**	**Sum of squares**	***df***	**Mean square**	***F***	***p***
Channel	280.96	26	10.81	12.00	<0.001
Time	2.63e−28	1	2.63e−28	2.92e−28	1.000
Channel * Time	23.37	26	0.90	1.00	0.466
Residuals	2285.67	2538	0.90		

**Figure 4.**
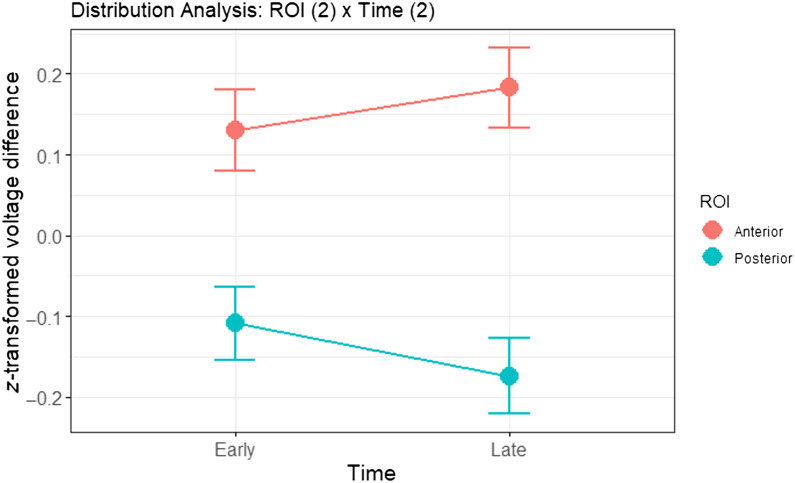
Visualization of *z*-transformed voltage difference between the no overlap and the predictable condition, averaged over channels in the ROIs for the ROI * Time (2 × 2) ANOVA.

The second ANOVA collapsed the Channel factor into the 2-level factor of ROI, using data from the channels in our preregistered anterior ROI (F3, F4, Fz, F7, F8, FCz, FC1, FC2, FC5, FC6) and posterior ROI (CP1, CP2, CP5, CP6, Pz, P3, P4, P7, P8). The results are presented in Table 5 and in Figure 4. Again, we do not observe a significant interaction between ROI and Time (*F*(1, 1724) = 1.438, *p* = 0.231). These results are in contrast to Van den Brink et al. (2001), but not Van den Brink and Hagoort (2004). Using the same analyses, the latter failed to observe the significant interaction reported by Van den Brink et al. (2001). Indeed, although there may be a trend toward an interaction in our data, the results we present here suggest that the interaction is less reliable than would be expected from some of the earlier reports.

**Table 5.** Results of the ROI(2) * Time(2) ANOVA on the *z*-transformed voltage difference between the no overlap condition and the predictable condition.

**Parameter**	**Sum of squares**	***df***	**Mean square**	***F***	***p***
ROI	37.90	1	37.90	37.04	<0.001
Time	2.90e−06	1	2.90e−06	2.83e−06	0.999
ROI * Time	1.47	1	1.47	1.44	0.231
Residuals	1764.27	1724	1.02		

### Exploratory Bayes Factor Analyses

The previous analyses involved null-hypothesis significance testing, which does not allow us to quantify the evidence for/against the ROI * Time interaction. We therefore conducted a Bayes factor analysis using the Bayes Factor package (Morey & Rouder, 2018). Crucially, we find moderate evidence for the addition model (i.e., ROI + Time) over the interaction model (BF = 6.5). In other words, we find moderate evidence against an interaction between ROI and Time, and therefore against a qualitatively distinct effect in the two time windows.

## DISCUSSION

In this EEG study on Dutch spoken sentence comprehension, we investigated the neural consequences of unpredictable words (e.g., *tonnetje* / *toetertje* (barrel / horn)) in sentences that presumably led participants to predict a highly predictable word (e.g., *boeket* (bouquet)). Specifically, we examined whether unpredictable words elicit an early negativity (N200/PMN) that is associated with phonological processing and distinct from the N400 component. We also examined whether this effect is influenced by the amount of phonological overlap in the (vowel of the) first syllable (for the predictable word *boeket*, there is no overlap in *tonnetje*, but partial overlap in *toetertje*).

Using preregistered analyses, we followed previous research (Van den Brink et al., 2001; Van den Brink & Hagoort, 2004) in comparing the scalp distributions of effects in an early time window (150–250 ms after word onset) and a later time window (300–500 ms after word onset). ERPs elicited by unpredictable and predictable words diverged as early as 150 ms post word-onset, consistent with previous findings (Nieuwland et al., 2020; Van Berkum et al., 2003; Van den Brink et al., 2001; Van Petten et al., 1999), with enhanced negative ERPs for unpredictable words (with or without partial overlap) compared to predictable words. Crucially, we did not obtain evidence that the scalp distribution of the predictability effect differed between the early and late time window, yielding no support for a distinct PMN effect (see also Van Petten et al., 1999). Moreover, our Bayesian analyses yielded moderate evidence *against* such a critical interaction between time window and scalp distribution. In sum, our results cast doubt on the existence of a distinct ERP component reported as evidence for phonological prediction.

### Failure to Replicate the PMN Effect

Our study was an attempt to replicate the PMN effect associated with phonological prediction (Connolly & Phillips, 1994) and the related N200 effect (Van den Brink et al., 2001; Van den Brink & Hagoort, 2004), using a participant sample size (*N* = 48) that was more than double that of some previous studies (*N* = 20). Unpredictable words in our study elicited more negative voltage than highly predictable words, both in the early time window associated with the N200/PMN and the later window associated with the N400. However, our results do not support the hypothesis that unpredictable words generated a PMN (or N200) effect, an effect previously associated with a pre-semantic phase of processing that purely deals with phonological information (Connolly & Phillips, 1994). Evidence for such an effect should have come for a distinct scalp topography in the early and late time windows. Although the obtained ERPs patterned in that direction, we did not obtain evidence *for* such an effect and, in fact, Bayesian analyses yielded moderate evidence *against* such a pattern. Therefore, our results call into question the reliability of a distinct early ERP component that is specific to phonological mismatch during spoken sentence comprehension. In all, although absence of evidence (for a phonology-specific ERP component) obviously does not equal evidence of absence, our findings are more compatible with the notion that the observed early negative ERP is in fact an early onset of the auditory N400.

A lack of a PMN effect has already been reported in similar paradigms (Diaz & Swaab, 2007; Van den Brink & Hagoort, 2004; Van Petten et al., 1999). Furthermore, several other studies have questioned the reliability of the PMN effect, in particular in the way that researchers have tried to identify this effect (e.g., Van Petten et al., 1999). A recent, critical review of the available literature by Nieuwland (2019) highlighted the different concerns regarding the PMN and the N200 effect, in particular that previous work defines the N200/PMN effect in different ways and observed N200/PMN effects in very differently appearing effects (see also Lewendon et al., 2020). Nieuwland concluded that previous work typically confused the N200/PMN effect with residual alpha noise (alpha activity that is not functionally related to the phenomenon of interest but that is nevertheless visible in the grand-average ERP waveforms), and suggested a more parsimonious account should be considered, namely that phonological deviations from expected words elicit rapid-onset N400 effects but no distinct N200/PMN effect (e.g., Van Petten et al., 1999).

The current study therefore further substantiates these previously raised concerns. Since similar paradigms produce variable results across studies, the effects in question may not be as reliable as previously suggested. Moreover, to our knowledge, our study is unique because it is the first explicit attempt at replication of the PMN prediction effect, it employs a much larger sample size than previous work, and it reports direct evidence *against* the critical interaction between time window and scalp topography.

### Effects of Phonological Overlap

Models of spoken word recognition, such as TRACE (McClelland & Elman, 1986) and Shortlist B (Norris & McQueen, 2008), implement a continuous mapping of acoustic input onto lexical representations. This is consistent with the results of eye tracking studies that show that listeners’ looks to different objects in the display change as speech unfolds. For example, the proportion of looks to onset-overlapping cohort words, such as *beetle* and *beaker*, are initially equal, until there is sufficient acoustic information in the speech to confirm which word is being said (Allopenna et al., 1998). Furthermore, the proportion of looks to images that correspond to rhyming words increases when the overlapping phonological information is presented (i.e., looks to *speaker* increase while *beaker* is being presented). This demonstrates that listeners consider multiple candidate words as more information is presented, and may continue to map incoming acoustic information to candidate lexical representations as speech unfolds.

From this, it follows that ERP effects in the early (PMN) time window could be influenced by acoustic information in the onset of an unpredictable word (e.g., *toetertje* (horn)) that overlaps with that of the predictable word (e.g., *boeket* (bouquet)). However, in contrast to this hypothesis, Newman et al. (2003) report that ERPs in the early time window reflect an all-or-none response to mismatch detection. Therefore, in order to test whether ERP amplitude was modulated by the amount of phonological overlap, we included the partial overlap condition (i.e., unpredictable *toetertje* when *boeket* was predictable).

The amount of overlap impacted ERPs at the anterior ROI in an unexpected way in our results, with more negative ERPs for partial overlap compared to no overlap, the opposite of what would be expected if this effect genuinely reflects a phonological mismatch detection. However, visual inspection of the ERP waveforms (Figure 2) suggests that this small effect is due to a sustained negative divergence that started before word onset. We therefore do not take this pattern as a genuine effect of phonological mismatch detection. Furthermore, the difference between the two unpredictable conditions is so small (less than half a microvolt) that even if this is a signature of a distinct early ERP component, it does not appear to be as clear cut as previous studies have indicated. In sum, our results do not support the hypothesis that phonological overlap with a predictable word, at least in the first syllable of a word, impacts the initial stages of word processing (see also Politzer-Ahles et al., 2022).

### Implications for Phonological Prediction

As we did not observe an early ERP component that was distinct from the N400, and we therefore cannot provide evidence for effects specific to phonological processing, one possible conclusion is that our results are most consistent with the purely semantic account of the N400. However, the results of other studies suggest that the amplitude of negative-going ERPs can also be influenced by phonological mismatch. For example, using a lexical decision task, Praamstra et al. (1994) found a reduced N400 amplitude to words that rhyme with a prime compared to non-rhyming words. Furthermore, Diaz and Swaab (2007) report that phonologically incongruent words presented following an alliterative list elicited a negative-going ERP component. This suggests that multiple processes that are sensitive to predictability violations at different levels of representation can contribute to the negativities observed in what we are considering to be a wider auditory N400 time window. Therefore, our results are also consistent with a multiple-processes account of the N400, one that considers violations of predictions at multiple levels of representation to be reflected in the N400 component.

At the same time, even though our results are also consistent with a purely semantic account of the N400, it is important to note that our findings do not rule out phonological prediction per se. The absence of the clear phonology-specific ERP component prevents us from drawing conclusions about phonological prediction and the pre-activation of phonological information during sentence processing. However, it may be that EEG and ERPs are not ideally suited to detect functionally distinct components. It is well known that EEG signals are measured over a wide area, and given that the method is susceptible to spatial smearing, EEG scalp topographies are a rather crude measurement of the underlying sources of electrical activity. A more spatially sensitive approach might be to use MEG. Indeed, Signoret et al. (2020) source-localized an early effect (which they label the MMN) to different dipoles than their observed N400. Therefore, other neuroimaging methods may be the way forward in studying the neural correlates of prediction. This, coupled with the fact that the ERP effects investigated in the present study have proven to be unreliable, indicates that skepticism concerning previous research is warranted, and conclusions about prediction and sentence processing that are based on these ERP effects may not be as robust as previously thought.

### Conclusion

Overall, our results do not support a functionally distinct ERP component that is specific to phonological processing. Instead, we see reason to interpret our results as an early onset to the auditory N400. This interpretation of the N400 is consistent with a purely semantic N400 account (i.e., where the early onset of the component reflects rapid mapping of acoustic-phonetic input to semantics) or, alternatively, a multiple processes account (i.e., where the detection of both phonological and semantic prediction violations underlie N400). However, the results of the current study cannot distinguish between these two accounts, and although distinct components such as the PMN and N200 are broadly cited in the literature as clear demonstrations of phonological mismatch detection, the findings of the present study indicate that skepticism about these components is warranted. Finally, our results do not dismiss phonological prediction during sentence processing. Rather, further research, including the use of various neuroimaging techniques, is needed to explore the extent of phonological prediction during sentence processing.

## ACKNOWLEDGMENTS

We would like to thank James McQueen for helpful discussion and comments on an earlier version of this manuscript. We would also like to thank Birgit Knudsen and Iris Smits for data collection.

## FUNDING INFORMATION

Victoria R. Poulton, Natural Sciences and Engineering Research Council of Canada (https://dx.doi.org/10.13039/501100000038), Award ID: PGSD-545923-2020.

## AUTHOR CONTRIBUTIONS

**Victoria R. Poulton**: Conceptualization; Data curation; Formal analysis; Investigation; Methodology; Project administration; Software; Visualization; Writing – original draft; Writing – review and editing. **Mante S. Nieuwland**: Conceptualization; Formal analysis; Funding acquisition; Methodology; Project administration; Resources; Supervision; Writing – original draft; Writing – review and editing.

## TECHNICAL TERMS

ERP component: A component wave of the ERP, defined by timing, scalp distribution, polarity (positive or negative amplitude), and response to different types of stimuli.
Event-related potential (ERP): Event-related voltage changes in the EEG signal that are time-locked to specific events.
Phoneme: An individual sound unit in a language, e.g., /p/ or /t/.
