# Supplementary material for: Can You Hear What’s Coming? Failure to Replicate ERP Evidence for Phonological Prediction
Source: Neurobiol Lang (Camb). 2022 Sep 22;3(4):556–74. doi: 10.1162/nol_a_00078 (PMC10158594; doi:10.1162/nol_a_00078)
Supplement: Supplementary file 1 [file nol-3-4-556-s001.docx]

**Supporting Information**

**Appendices:**

**A. Grand-average ERPs for the predictable, partial overlap, and no overlap conditions.**

**
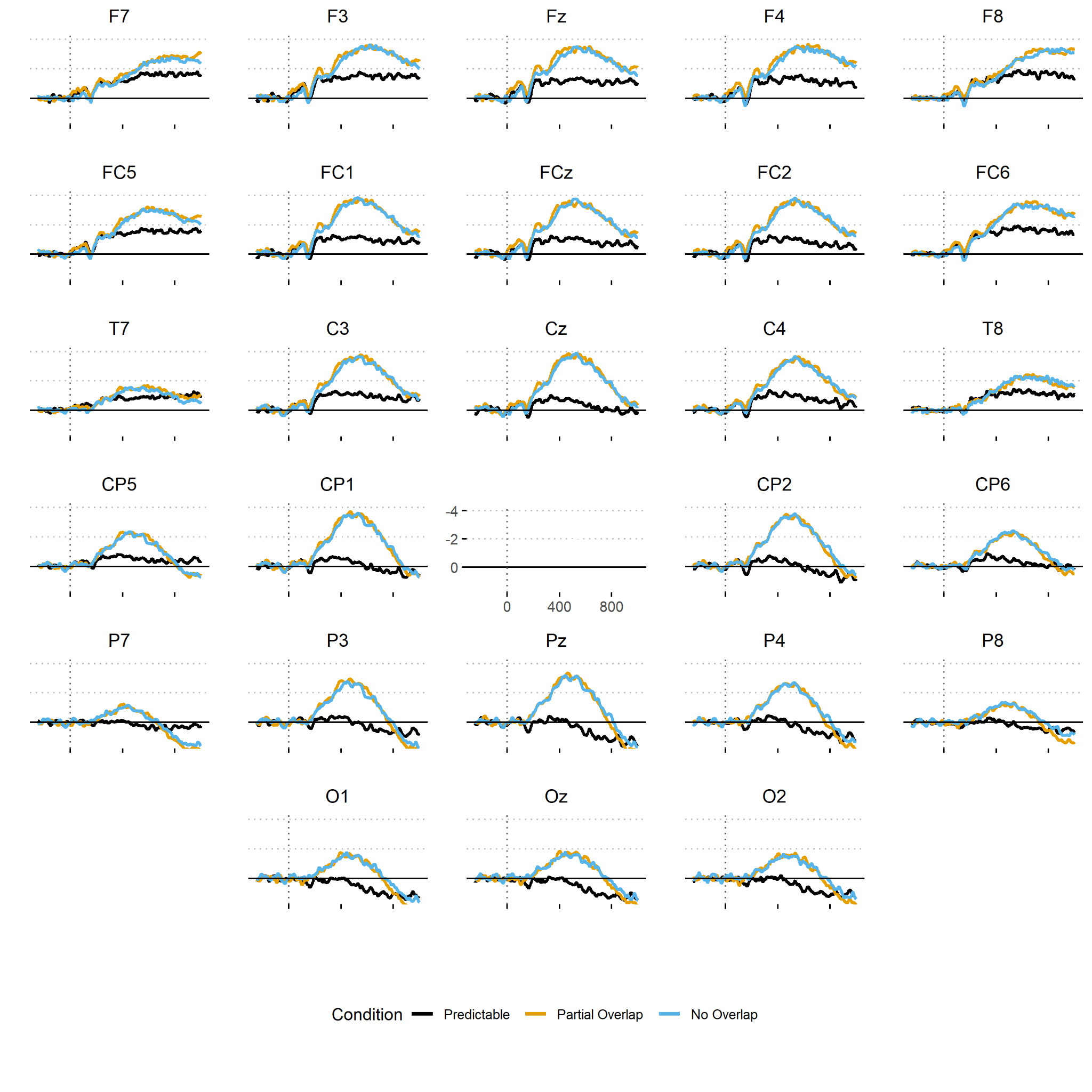
**

**B. Detailed results of the linear mixed effect regression**

**Table B.1:** LMER results for the early time window (150-250ms). Confidence level = 95%. Significant p-values are indicated in bold (<0.05).

| **Parameter** | **Coefficient** | **SE** | **95% CI** | **t(18749)** | **p** |
| --- | --- | --- | --- | --- | --- |
| (Intercept) | -0.65 | 0.08 | (-0.81, -0.48) | -7.66 | **< .001** |
| Condition1 (No Overlap) | -0.04 | 0.06 | (-0.17, 0.08) | -0.64 | 0.520 |
| Condition2 (Partial Overlap) | -0.25 | 0.06 | (-0.38, -0.13) | -3.91 | **< .001** |
| ROI1 (Anterior) | -0.25 | 0.05 | (-0.34, -0.16) | -5.47 | **< .001** |
| Condition1 * ROI1 | 0.09 | 0.06 | (-0.03, 0.22) | 1.44 | 0.148 |
| Condition2 * ROI1 | -0.02 | 0.06 | (-0.15, 0.10) | -0.33 | 0.739 |

**Table B.2:** LMER results for the late time window (300-500ms). Confidence level = 95%. Significant p-values are indicated in bold (<0.05).

| **Parameter** | **Coefficient** | **SE** | **95% CI** | **t(18749)** | **p** |
| --- | --- | --- | --- | --- | --- |
| (Intercept) | -1.76 | 0.12 | (-1.99, -1.52) | -14.55 | **< .001** |
| Condition1 (No Overlap) | -0.41 | 0.07 | (-0.54, -0.28) | -6.06 | **< .001** |
| Condition2 (Partial Overlap) | -0.57 | 0.07 | (-0.70, -0.44) | -8.43 | **< .001** |
| ROI1 (Anterior) | -0.27 | 0.05 | (-0.36, -0.17) | -5.59 | **< .001** |
| Condition1 * ROI1 | 0.13 | 0.07 | (2.72e-03, 0.27) | 2.00 | **0.045** |
| Condition2 * ROI1 | 0.07 | 0.07 | (-0.07, 0.20) | 0.97 | 0.330 |

**C. Results of the Bayes Factor analysis**

**Table C.1:** Bayes Factor (BF) analysis of the ROI*Time (2x2) ANOVA. A BF <1 indicates evidence for the null hypothesis, while a BF >1 indicates evidence for the alternative hypothesis. BFs were calculated against an intercept-only model in the denominator.

|  | Bayes Factor | Proportional Error |
| --- | --- | --- |
| Time | 0.05404132 | ±0.05% |
| ROI | 4285965 | ±0% |
| Time + ROI | 224897.1 | ±2.17% |
| Time + ROI + Time:ROI | 34425.15 | ±2.08% |
|  |  |  |

**ANOVA models vs. the ROI*Time model**

**
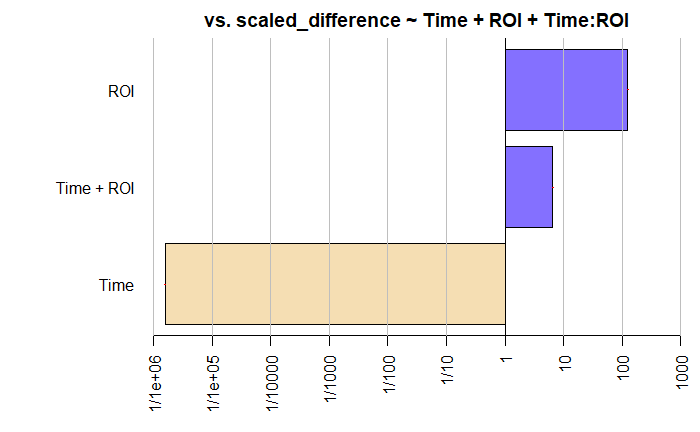
**

**Figure C.1:** A comparison of the Bayes Factors for the simpler ANOVA models (Time-only, ROI-only, and Time+ROI) against the interaction model (ROI*Time). The horizontal axis is plotted with a log-scale. Purple bars indicate Bayes Factors greater than that for the interaction model, while the yellow bar indicates a Bayes Factor less than that for the interaction model. It is clear that the evidence is greater in support of the Time+ROI and ROI only models over the interaction model.
